# Rattlesnake migrations and the implications of thermal landscapes

**DOI:** 10.1186/s40462-020-00202-0

**Published:** 2020-05-27

**Authors:** Jessica A. Harvey, Karl W. Larsen

**Affiliations:** 1 Environmental Science Program, Thompson Rivers University, Kamloops, Canada; 2 Victoria, Canada; 3 Department of Natural Resource Science, Thompson Rivers University, 805 TRU Way, Kamloops, British Columbia V2C 0C8 Canada

**Keywords:** Thermal ecology, *Crotalus oreganus*, Ectotherm, Incident solar radiation, Thermoregulation, Habitat selection, Random walk, Migration strategies

## Abstract

**Background:**

The importance of thermal resources to terrestrial ectotherms has been well documented but less often considered in larger-scale analyses of habitat use and selection, such as those routinely conducted using standard habitat features such as vegetation and physical structure. Selection of habitat based on thermal attributes may be of particular importance for ectothermic species, especially in colder climates. In Canada, Western Rattlesnakes (*Crotalus oreganus*) reach their northern limits, with limited time to conduct annual migratory movements between hibernacula and summer habitat. We radio-tracked 35 male snakes departing from 10 different hibernacula. We examined coarse-scale differences in migratory movements across the region, and then compared the route of each snake with thermal landscapes and ruggedness GIS maps generated for different periods of the animals’ active season.

**Results:**

We observed dichotomous habitat use (grasslands versus upland forests) throughout most of the species’ northern range, reflected in different migratory movements of male snakes emanating from different hibernacula. Snakes utilizing higher-elevation forests moved further during the course of their annual migrations, and these snakes were more likely to use warmer areas of the landscape.

**Conclusion:**

In addition to thermal benefits, advantages gained from selective migratory patterns may include prey availability and outbreeding. Testing these alternative hypotheses was beyond the scope of this study, and to collect the data to do so will require overcoming certain challenges. Still, insight into migratory differences between rattlesnake populations and the causal mechanism(s) of migrations will improve our ability to assess the implications of landscape change, management, and efficacy of conservation planning. Our findings suggest that such assessments may need to be tailored to individual dens and the migration strategies of their inhabitants. Additionally, local and landscape-scale migration patterns, as detected in this study, will have repercussions for snakes under climate-induced shifts in ecosystem boundaries and thermal regimes.

## Background

The conservation of migratory animals may be particularly challenging, as movements may cross multiple habitats and jurisdictions, including borders between protected and unprotected areas [58]. A starting point is determining habitat use patterns throughout the entire life-history of these species, followed by an understanding of why these patterns exist, and why particular migratory paths are followed. Such information is critical to crafting effective conservation and management strategies.

Migrations occur when animals move explicitly to take advantage of resources distributed through the environment [20, 23, 35, 66]. Migration is differentiated from dispersal movements by way of bidirectional movement, movement of greater duration than ranging, and movement along relatively straight-line paths [20]. Such responses may be apparent when they involve clear, contrasting shifts in habitat use, such as birds migrating between temperate and tropical regions. However, migrations also occur on a finer landscape scale, where habitat factors exert more subtle effects. Food, water or mate availability may drive such movements [2], but for animals inhabiting cooler regions, heat potentially may be another resource influencing migration. Ectothermic animals occupying relatively harsh or variable environments may be particularly responsive to the thermal properties of landscapes, given the lack of a metabolic buffer between the thermal environment and the physiological function of the individual. Although there is consensus that a prominent factor in habitat selection by ectotherms is temperature [7, 17], this relationship has been primarily studied on a fine-scale [11, 17, 33, 64, 71]. Snakes, particularly northern species, provide rare examples of terrestrial ectotherms conducting annual migrations; these migrations can include considerable movements relative to the mobility of the animal and the brevity of the active season (e.g., [36, 47, 73]). However, the potential linkages between these movements and thermal landscape patterns has not been well-studied [72]. By testing for this mechanism, we gain a better understanding of why particular movements (including seasonal migrations) occur. This, in turn, will facilitate more refined habitat mapping and consideration of how other land uses could potentially influence migrations.

Seasonal, relatively lengthy migrations (> 3–5 kms) have been documented for the Western Rattlesnake (*Crotalus oreganus*) in areas close to the species’ northern limits [10, 29]. Presumably, as in other northern snakes, these movements are partly due to the geographic separation of suitable overwintering hibernacula (‘dens’) and summer habitat conducive to foraging and mating [31]. However, exactly why these animals conduct extensive movements is unclear, particularly when the animals are travelling through areas that contain prey. Further, the extent and directionality of the migratory movements appear highly den specific, with adult individuals taking similar pathways from year to year and, in at least one study, the animals may travel into higher-elevation forests away from the normally associated grassland habitat [29]. While den sites may be afforded protection through land designations (parks, protected areas, etc.) or stewardship by private land owners, migration routes can result in snakes travelling across different jurisdictional boundaries and into areas where protection is not afforded [85]. At present, we lack a clear understanding of why these animals use lengthy and specific migration routes during the summer active season.

Ectotherms, like snakes, employ strategies to maintain their body temperatures at optimal levels. These strategies include physiological mechanisms for temperature control and/or behavioural tactics. Behavioural thermoregulation carries ecological benefits and costs [77]; for example, shifting positions to maintain optimal body temperatures [28, 37, 84] will be worthwhile for an animal only the availability of other resources does not plummet [38] such as food and access to mates [7, 22]. Thermoregulation thus may be considered a form of ‘resource utilization’ [39] and variation of heat as a resource across different habitats likely will have repercussions [76].

Hypothetically, variations in movements and habitat use exhibited by different den populations of rattlesnakes and other northern snakes may be caused by temperature selection at the landscape level. Thermal characteristics on the ground will be influenced by the incident solar radiation that, in turn, is influenced by substrate ruggedness, day length, topographical shadows, and solar azimuth at the given latitude [40]. On an annual timescale, seasonal and daily variation in these dynamics likely do not cause major changes in vegetation communities typically used to delineate habitats; however, they may provide significantly different resources (from a snake’s point of view) at different points in the season and across a heterogeneous landscape. Over the course of the active season, the average thermal properties of the landscape may alter the migratory and large-scale movement patterns of snakes as they leave and return to hibernacula. If snake migration can be linked to thermal attributes of landscapes, it would provide powerful new insight into how the ecology of these animals is influenced on large scales. Being able to predict such movements also would be an important conservation tool.

The goals of this study were (i) to use telemetry to monitor the seasonal migration of Western Rattlesnakes from a relatively large sample of hibernacula across the northern range of the species, (ii) demonstrate more clearly the existence of different migratory phenotypes and associated habitat types, and (iii) examine the role that thermal attributes of the landscape play in large-scale habitat selection. Our working hypothesis was that snakes select warmer areas within the available habitat (‘landscape’) to gain thermoregulatory benefits over the course of the northern summer. We used solar insolation as a proxy for temperature; this metric has been used to predict hibernacula locations [32] but it has not been applied to investigate snake summer habitat use. Thus, we examined whether thermal properties, like other long-term resource distributions, are correlated with habitat use by animals. We conducted our work at the northern limit of rattlesnakes (western Canada), where harsher and more variable temperatures intuitively should cause the thermal attributes of landscapes to exert a relatively strong influence on seasonal migratory movements.

### Study species

The Western Rattlesnake (*Crotalus oreganus*) is the northernmost viper (F. Viperidae) in the Western Hemisphere, reaching lengths just over 1.2 m in snout-vent length [3]. The species’ range extends across the USA:Canada border into the southern region of British Columbia (BC, Canada), where the snake feeds primarily on rodents and birds [56]. In BC, the species occupies a limited and narrow range, being restricted primarily to the dry semi-arid valleys of the Okanagan and Thompson rivers (see map in [54]). Here, the snakes den communally, displaying high fidelity during their life to specific hibernacula typically located in south-facing rocky crevices, fissures and talus slopes in low-elevation (500–625 m above sea level [MSL]) grasslands [5, 51, 52]. After the snakes emerge from hibernacula in the spring (April, May), males disperse for the summer [53] presumably to feed and locate mates, and then return to the den in autumn (September, October). The movement ecology of females is far less studied, but reproduction is known to be biennial or longer, and there is some evidence that nongravid females also disperse from the den site for summer [52]. Mating takes place away from dens in late summer (July, August) with fertilization delayed until the ensuing spring. As embryos develop, the gravid females remain close to the den site and occupy ‘rookeries’ through gestation and parturition (Larsen pers. obsv.). The lower-valley locations of most hibernacula have led to these northern snakes historically being associated with grassland habitats during the entire active season; however, telemetry work by Gomez et al. [29] revealed that the snakes in one denning population migrated into higher-altitude forests, moving up to 3500 m from the hibernacula over the course of the summer, while snakes from a neighbouring den population remained primarily in the grasslands. Other studies have referenced occasional forest habitat use [10, 13, 14, 29, 50], suggesting the stereotyped habitat association of the species needs further investigation*.* In particular, the extent of migratory movements by this animal, and why they vary between different hibernacula populations, is a significant gap in our understanding of the species’ ecology in the far north.

## Methods

### Selection of sites

**Fig. 1:**
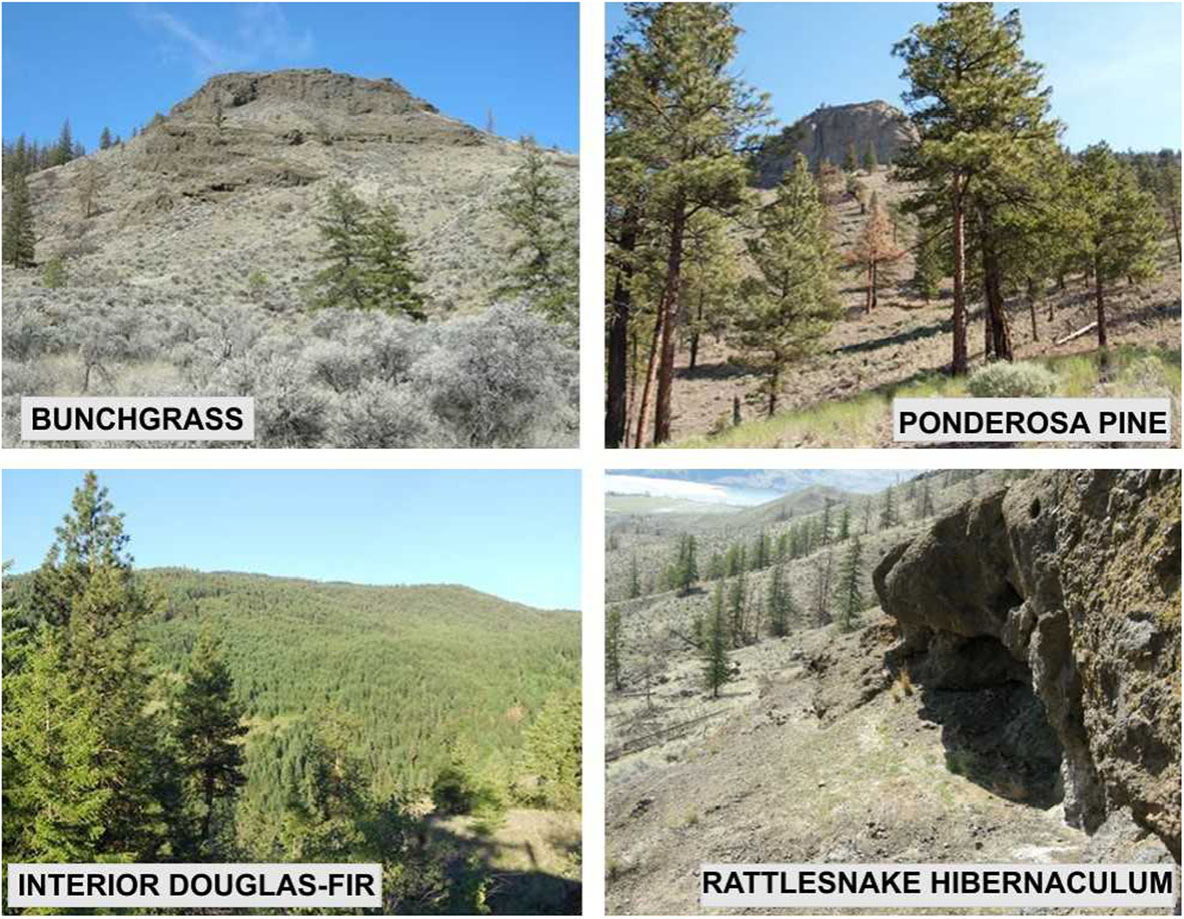
Typical and atypical habitat associations for the Western Rattlesnake in British Columbia. Open grassland habitats generally occur at elevations of 300 to 800 m in the Bunchgrass and Ponderosa Pine biogeoclimatic zones. Forested habitats generally occur at elevations of 500 to 1200 m in the Interior Douglas-fir biogeoclimatic zone [57]. Hibernacula typically occur at elevations of 500 to 625 m on south-facing, rocky slopes. Photos by lead author

The study was conducted in 2010 and 2011 in the Thompson-Nicola (50.8°N, 120.6°W) and Okanagan-Similkameen (49.3°N, 119.6°W) regions of British Columbia, Canada. This is a semi-arid region containing the northernmost populations of this species, with an elevational range from approximately 300 to 1400 MSL. Details on the ecosystem characteristics are provided in Fig. 1 and in Harvey [34]. Study hibernacula were selected to ensure a diversity of putative thermal landscape conditions were present between hibernacula. This was done using preliminary thermal maps of the area created with GIS (details below). Considerations also were made for logistics, land ownership and access. Further, hibernacula were only considered for the study if the estimated population size was > 12 snakes [9]; this was done to reduce the likelihood that the study would impact the viability of the population. In total, ten different hibernacula were chosen for this study – six in the Thompson-Nicola region (six in 2010, with one repeat in 2011) and four in the Okanagan-Similkameen region (in 2011).

In total 17 snakes from six hibernacula were selected for inclusion in the study in 2010, and 18 snakes from five hibernacula were selected in 2011. We targeted at least three snakes from each hibernaculum in order to achieve replication. However, at two hibernacula only one and two snakes respectively were deemed suitable for the study (based on length and weight). At the remaining nine sites, either three or four snakes were selected as study animals.

### Animal capture, processing, selection and surgery

We visited targeted hibernacula repeatedly (i.e., at least twice) during the spring emergence period (April 15 – May 7). Rattlesnakes were captured using tongs and weighed in a canvas bag. Individuals suitable for telemetry (i.e., heavier than 400 g) were ushered into a plexiglass tube for sex determination via hemipenal probing [75]. Because females vary in their migratory behaviour according to their reproductive status (see above - [51]), we selected only male snakes for radio-telemetry. All animal selected for telemetry were transported by vehicle to a veterinary clinic in a towel-lined, aerated rubber container.

### Radio-telemetry

Each study animal was surgically implanted with an SB-2 radio-transmitter (Holohil Systems Inc., Ontario, Canada), weighing 3.8–5.2 g. No implant package exceeded 2.7% of the snake’s weight, and transmitter lifespans ranged from 5 to 10 months. Surgeries were carried out by veterinarians following the protocols described by Reinert and Cundall [67] with modifications by Reinert [68]. Implanted snakes were held for approximately 24–48 h post-operation to permit recovery from sedation and allow rehydration. Each animal then was released at its precise point of capture. We tracked and located telemetered snakes every three to 7 days between egress (April/May) and ingress (September/October) using an R-1000 telemetry receiver (Comm-Spec) and RA-159 handheld Yagi directional antenna (Advanced Telemetry Systems). When each snake was located, we recorded date, time, UTM coordinates using a handheld GPS unit (Garmin, GPS 76Cx, accuracy ±3 m), canopy closure using a spherical crown densitometer (Forestry Suppliers, Convex model A), and habitat type (see below). Transmitters were removed from re-captured snakes either when they returned to their hibernacula in autumn of the same year, or as they emerged from hibernation the following spring.

### Mapping and analysis

Snake location data were filtered to include only those that constituted independent movements, defined as more than 10 m from the previous location [29]. Each location was assigned to one of two habitat types to enable comparison: those with < 10% canopy closure (bunchgrass and open-canopy Ponderosa Pine) were designated as “Open” habitats, while locations with > 10% canopy closure (Interior Douglas-fir forests) were classified as “Forest” habitat. In the Kamloops region, this threshold for “Forest” was deemed appropriate given the arid nature of the region and the typical canopy closure in the Ponderosa Pine zone. Additionally, each snake was later assigned a category based on the type of habitat reached at the endpoint of its migration, termed the ‘destination habitat’. This classification of habitats allowed comparison between snakes using typical (open grassland) habitats and atypical (forested) habitats (Fig. 1).

The migration path for each individual snake was created by connecting telemetry data points, assuming direct movements between locations. The maximum straight-line distance travelled by each snake from its hibernaculum was calculated using the furthest detected location (apogee). Also, we focused our movement analyses largely on the migration path taken by each snake out to the apogee, terming this the Outward migration path [rattlesnakes tend to simply reverse their Outward migration paths during the ‘homeward’ migration phase [29] when movements appear largely driven by fidelity to the den site]. The Outgoing migration was further divided into an Initial stage and a Late stage containing the movements of each snake in May/June and July/August, respectively.

**Fig. 2:**
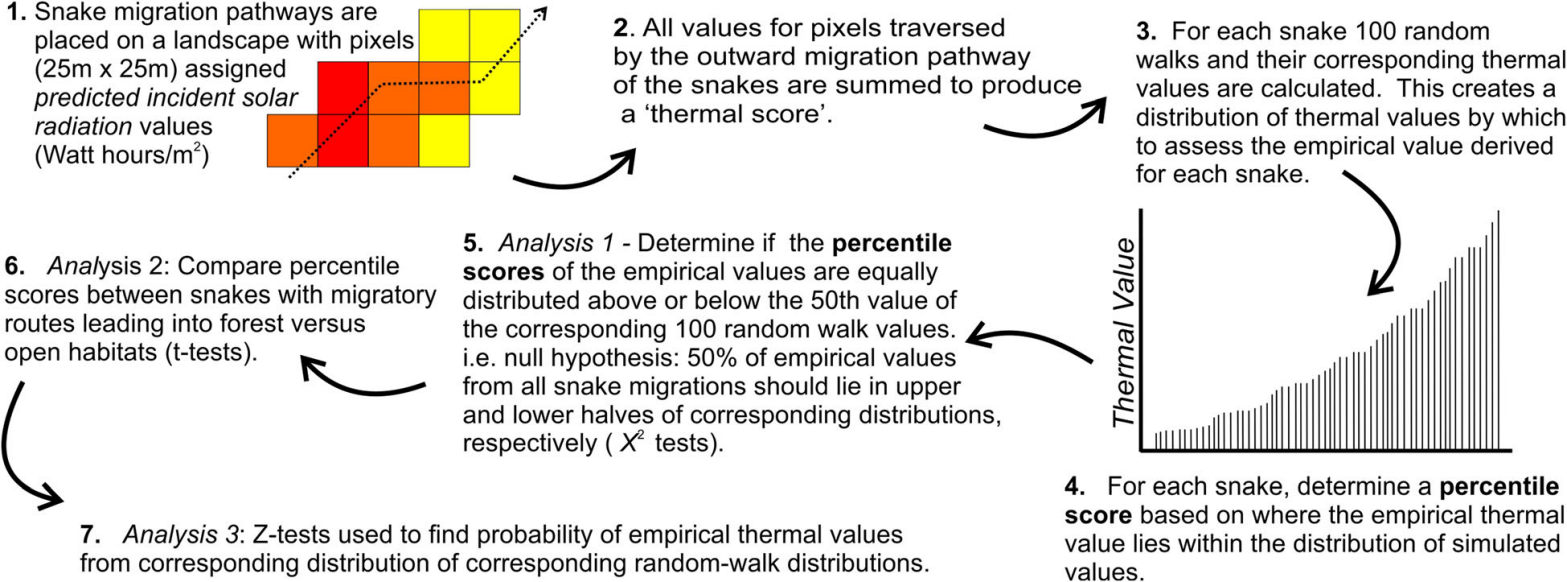
Step-by-step process by which assessment of the thermal properties of migration routes was conducted for rattlesnakes in British Columbia, Canada. The entire process outlined was separately carried out on the entire Outward migration pathway, as well as separately on the Initial and Late stages of the same migration pathway. Definitions and additional details are provided in the Methods section of the text

**Fig. 3:**
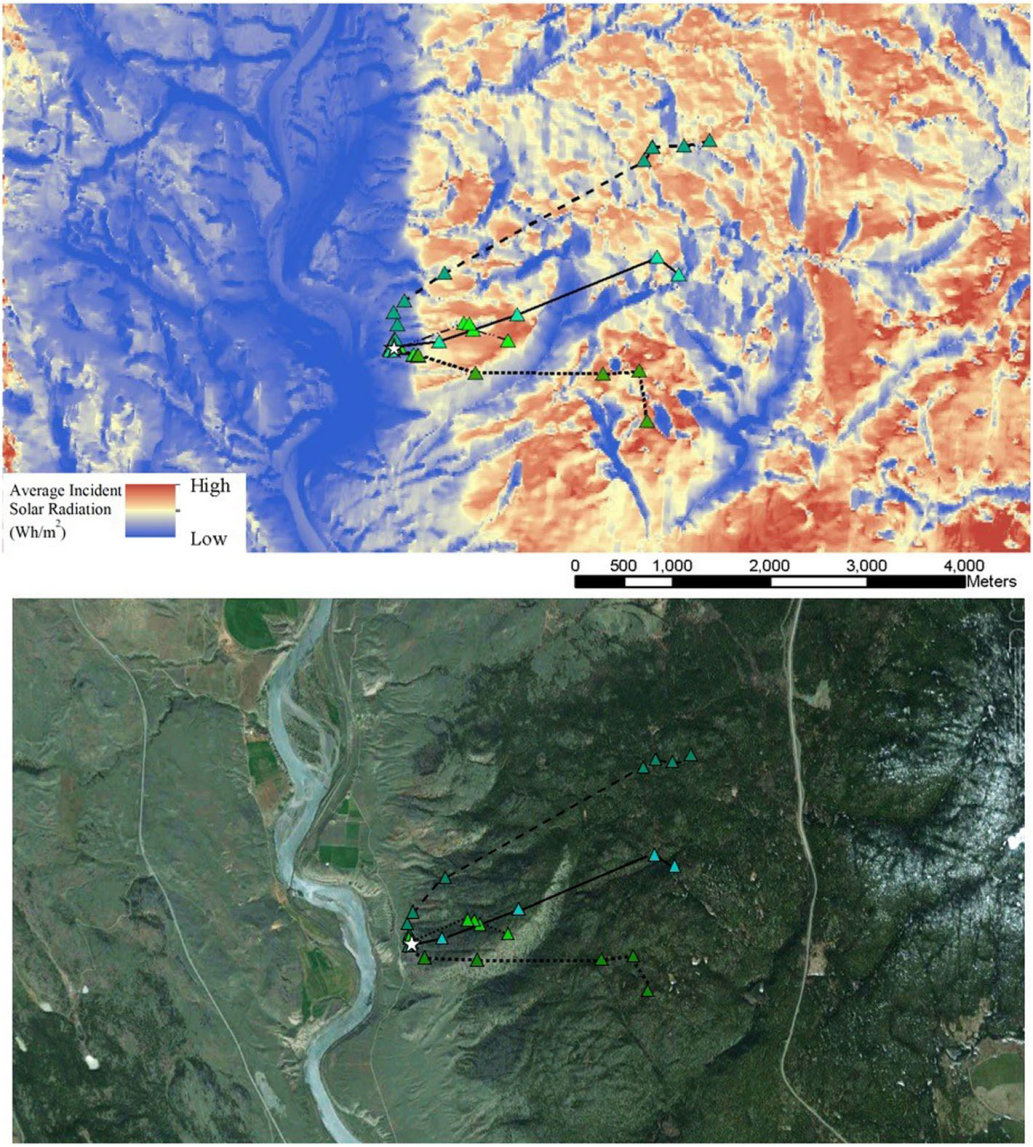
Examples of Western Rattlesnake migrations from the hibernaculum to destination habitat. In the top illustration, the study hibernaculum is represented by a star (☆), and different snake movements are represented by different-coloured triangles (Δ) and connecting lines. In the top image, the thermal landscape is represented using modelled average incident solar ration as a proxy for temperature. Telemetered snakes in this example all used warmer and forested areas of the thermal landscape (shown as darker green in the bottom image)

Our first step (detailed in Fig. 2) was to quantify the amount of solar radiation each migratory path would receive in an average year. To do this, we created thermal models of the landscapes surrounding the hibernacula from whence the telemetered snakes originated. These landscape simulations were composed of raster images built on the predicted incident solar radiation for a pixel (cell size) of 25 m^2^. To quantify the solar radiation each pixel would receive, we used the Area Solar Radiation tool in the Spatial Analyst extension in ArcGIS 9.3 [25]. This algorithm uses slope, aspect, elevation, day length, latitude and solar azimuth to calculate the expected incident solar radiation at a given point on the landscape. The simulation was based on a 25 m digital elevation model (DEM), with the thermal values being expressed in average daily Watt-hours per square meter (Wh/m^2^) (Fig. 3). It is important to appreciate that these maps did not depict the actual solar radiation a snake would experience during any given year; rather, the modelling process quantified the level of solar radiation (i.e. temperature) each pixel would be expected to receive *on average* across years, based on general topographic features of the landscape [8, 21, 27].

Our second step (Fig. 2) was to produce a ‘thermal score’ for the migratory routes demonstrated by the snakes. To do this, we calculated the total of the solar incidence values assigned to those pixels traversed by each snake’s Outward migration path. We also calculated these values for the Initial and Late portions of the migratory pathways.

To examine the significance of the pathway thermal values, we used a relatively simple analytical approach, given that more elaborate and sophisticated step-selection functions (SSF - [79]) target situations where massive amounts of sequential spatial data are collected on animals, primarily through GPS (e.g. [62]), other remote tracking methods, or constant tracking [80] – all methods we could not employ in this study. These SSF models become more difficult to parameterize and interpret when relocation frequencies are low and vary considerably between animal subjects, such as the situations we faced in our study (see Results). Further, to our knowledge, this type of study has not appeared in the literature, and more complex analyses could make the results more difficult to understand while likely leading to similar conclusions [59].

Our analytical approach started with the creation of random-walk migrations for each of the snakes we tracked (see Step 3, Fig. 2). This allowed comparisons involving our empirical/observed thermal values to those theoretically ‘available’ on the landscape, thus representing a form of resource use [44]. We used Hawth’s tools [6] to generate 100 random walks for each snake, starting at the animal’s hibernaculum. These random walks were parameterized using the actual Outward movements of the snake: i.e. the overall lengths of the random walks were restricted in length to the greatest distance measured from the hibernaculum to the snake (the apogee), and an average turn angle equal to that demonstrated by the animal in the field. For each random walk, we calculated a thermal value for the simulated pathway in the same manner as that previously done for the empirical (observed) movement pathway (see above). Repeating this procedure for the 100 simulated random walks by the snake produced a distribution of thermal values by which to compare the empirical value. From there, we simply determined the percentile score of the observed thermal value within the distributions of values obtained from the random walk simulations (Step 4, Fig. 2). We repeated these calculations on the Initial and Late stages of the migrations. The end result was percentile values reflecting the Outward migrations of the sample of telemetered snakes (*n* = 30, see Results), with similar datasets derived from the Initial and Late stages of the migrations.

Our statistical analysis was three-fold: We first tested whether the observed thermal values for the snakes were relatively high or low given the landscapes they were migrating through. We simply noted whether they fell above or below the 50th percentile scores in the corresponding distribution of simulated values and applied a χ^2^ test H_o_: 50% of observed observations will fall above the 50th percentile of the 100 simulations (Step 5 – Fig. 2). We then tested if the percentile scores assigned to the snakes differed between animal using forest versus open destination habitats, using a t-test ([41, 88]- Step 6 – Fig. 2). Lastly, Z-tests [87] were used to determine the probability of selecting the observed thermal value of each snake’s migration from the distribution of values drawn from the corresponding simulated walks (Step 7 – Fig. 2). All of these analyses were repeated for each of the three divisions of snake migration (Outgoing, Initial, and Late). Normality of data were confirmed in all cases prior to analyses.

We also conducted an analysis on the role of ‘ruggedness’ in influencing the snake movements. Ruggedness as used in GIS environments in based on differences in elevation between a focal pixel and the eight pixels surrounding it [69]. Using Relative Topographic position and the raster calculator tool, each of these 8 differences is squared (to create all positive values) and a mean value is calculated. The square root of this value then produces the ruggedness index value [42] representing the average elevation change between the focal pixel and the neighbouring area. A ruggedness index value was calculated for each pixel on the landscape using a moving window analysis [42, 69]. The mean value of these pixels for each of the snakes’ outgoing migration paths and for an area with a radius of 4000 m surrounding each hibernaculum was calculated. Ruggedness could theoretically influence snake movements in two manners: (i) ruggedness could impact the mobility and capabilities of a snake to traverse the area, and/or (ii), a high ruggedness value may represent a diversity of microclimates existing on the site, allowing a snake to locate warm areas even on sites where the superficial thermal index is low.

Lastly, to determine the relationship between habitat ruggedness, thermal landscape characteristics, and habitat use, we examined the data in two ways. We first tested for a relationship between the two categories of snakes based on their destination habitat (forest versus open) using a t-test. We then used linear regression to explore the relationship between ruggedness index and the percentile scores calculated for the thermal values of each snake’s migratory pathway.

### Statistical considerations

All statistical analyses were performed in the program R version 2.12.1 [65]. Data were tested for normality by examination of histograms and using the Shapiro-Wilk test or the Kolmogorov-Smirnov test [87]. Homogeneity of variances between groups was tested using the Fligner-Killeen test [15, 16]. Percentage data were transformed for normality using an arcsine transformation for analysis. A significance value of α = 0.05 was used to guide the interpretation of the results. Means are reported ±1 standard deviation, unless otherwise stated.

## Results

In total, we used 35 snakes for the radio-telemetry study. Thirty of 35 telemetered snakes were tracked through their entire annual migration, resulting in 13 to 27 location data points per individual. Five snakes did not have their entire migration route completely documented either due to mortality or signal loss. These snakes were not considered in the analysis due to incomplete data sets.

In total, the migration data from 30 snakes were included in the analysis. As expected, the snakes travelled away from the hibernaculum to summer habitats (Outgoing migration), reaching their apogee on an average date of August 8. The earliest apogee occurred on June 23 and the latest on September 21. Most snakes returned to the hibernaculum (Homeward migration) along a very similar path as their Outgoing migration. The mean maximum straight-line distance measured for Outgoing migration (from the hibernaculum to the apogee) of the telemetered snakes was 1847.8 m ± 930.0 m (*n* = 30, range = 373.0 m to 3985.7 m). The migration metrics are presented in Table 1. There were no significant differences observed between straight-line migration distances between the Thompson-Nicola and Okanagan-Similkameen regions [migration (t_26_ = 1.42, *P* = 0.170).

**Table 1:** Distances and trends of movements by Western Rattlesnakes radio-tracked from ten hibernacula in British Colombia (2010 and 2011). The movements of snakes originating at the same den were considered ‘Directional’ if all snakes tracked from the hibernaculum travelled trajectories within a 40° range of each other and ‘Random’ if the trajectories ranged over more the 40°. The ruggedness index was calculated for an area surrounding each hibernaculum using Relative Topographic position. Means are reported ±1 standard deviation, unless otherwise stated

Region	Code name, Hibernacula	Number of study snakes	Mean maximum straight-line distance (m)	Movement directionality^1^	Destination habitat types (number of study snakes)	Ruggedness Index^1^
Thompson-Nicola	TN1	3	2165 ± 554.0	Directional	Forest (3)	76.3
	TN2	1	1478	n/a	Open (1)	60.5
	TN3	3	1908 ± 200.1	Directional	Open (3)	38.1
	TN4	4	2822 ± 1180.1	Directional	Forest (4)	62.9
	TN5	2	1293 ± 42.9	Random	Open (2)	58.4
	TN6	3	1854 ± 966.5	Random	Forest (2); Open (1)	43.4
Okanagan-Similkameen	OS1	4	1062 ± 574.1	Random	Open (4)	74.8
	OS2	3	738 ± 481.7	Random	Forest (1); Open (2)	45.7
	OS3	3	2420 ± 841.8	Random	Forest (2); Open (1)	68.0
	OS4	4	2143 ± 1018.1	Directional	Forest (3); Open (1)	58.8

All study hibernacula (and thus the starting points of all monitored migrations) were located in open habitats. From these sites, the mean distance to forest habitat was 656 m (± 958 m). Fifteen of the 30 snakes, henceforth termed ‘Forest snakes’, travelled to and used forests as a Destination habitat (i.e., in the latter part of their outgoing migrations during July and August), while the use of open habitats through the entire migration was observed in the other 15 study snakes (henceforth termed ‘Open-Habitat snakes’ - see Table 1). Maximum straight-line distances reached from the hibernaculum were significantly longer for Forest snakes (2359 m ± 837.0 m) than Open-Habitat snakes (1337 m ± 729.0 m; t_27_ = 3.57, *P* = 0.001) for the entire season.

Due to the limited sample size of snakes at each study site (<four from the majority of the study hibernacula), statistical analysis of migration directionality by the snakes from each hibernaculum was not possible. We, therefore, used a less-rigorous approach by classifying migrations from a particular hibernaculum as directional when the telemetered snakes leaving that hibernaculum displayed mean migration bearings within 40° of one another (Table 1). Within-hibernaculum groups of snakes whose migration bearings were more than 40° from one another were considered to have a random distribution. This distinction was made based on a natural break in the data and qualitative judgement of snakes’ travel directions.

The observed thermal metrics of the Forest snakes tended to occur in the upper half of the distribution of simulated migration paths (i.e., relatively warmer pathways) more often than Open-Habitat snakes, in all three categories of the migration (Outgoing, Initial and Late stages; see Fig. 3, Table 2A).

**Table 2:** Comparisons of observed thermal metrics to the thermal metrics derived from 100 random-walk migration path simulations for Western Rattlesnakes in British Columbia. Using thermal landscape maps, both observed thermal metrics and those of the simulated migrations were derived from the average incident solar radiation along the migration path for each migration category (Outgoing, Initial and Late stages). Forest snakes utilized forested habitats as the destination for their migration, while Open-Habitat snakes remained in sparsely-treed or open grasslands throughout the active season. Means are reported ±1 standard deviation, unless otherwise stated

Comparison	Group	Outgoing Migration	Initial Stage	Late Stage
A. Proportion of snakes occurring in the upper half of the distribution of the simulated migration paths	Forest snakes	13/15	11/15	14/15
	Open–Habitat snakes	8/15	5/15	8/15
		χ^2^_1_ = 3.9, *P* = 0.046	χ^2^_1_ = 4.8, *P* = 0.028	χ^2^_1_ = 6.1, *P* = 0.013
B. Average percentile scores of migration path values within values derived from the simulated migration paths	Forest snakes	76.6 ± 25.4	68.5 ± 28.7	79.3 ± 18.8
	Open–Habitat snakes	51.1 ± 22.0	43.7 ± 18.0	50.2 ± 26.1
		t_27_ = 2.95, *P* = 0.006	t_21_ = 3.03, P = 0.006	t_28_ = 3.46, *P* = 0.002
C. Average probabilities (as determined by z-test scores) of migration path values as tested against a distribution of values derived from the simulated migration paths	Forest snakes	0.24 ± 0.28	0.29 ± 0.26	0.25 ± 0.26
	Open–Habitat snakes	0.54 ± 0.30	0.68 ± 0.20	0.55 ± 0.27
		t_28_ = −2.66, *P* = 0.013	t_26_ = −4.65, *P* < 0.001	t_28_ = −3.17, *P* = 0.004

**Fig. 4:**
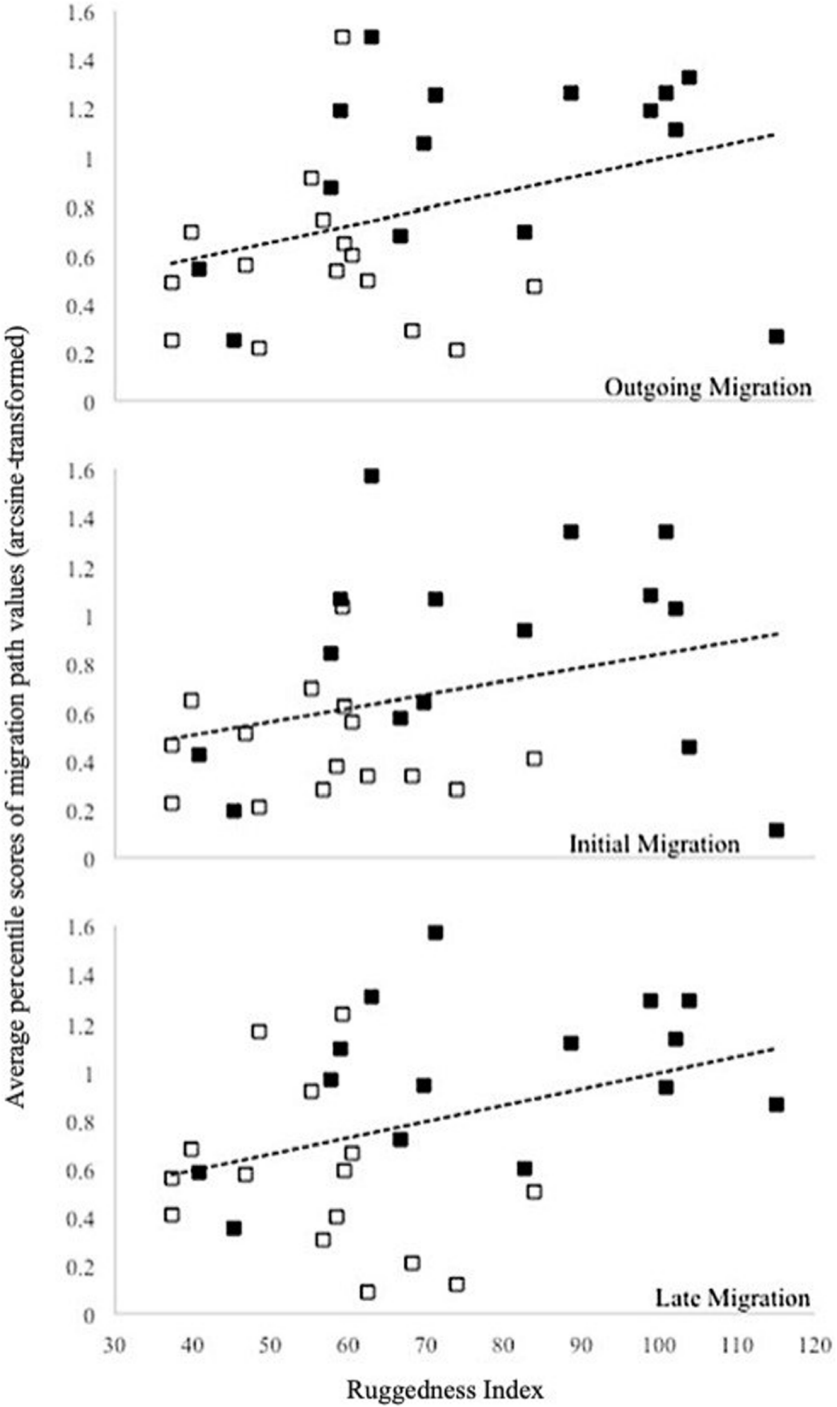
Average percentile scores of migration path values within values derived from 100 random-walk simulations, for each category of migration, compared to migration path ruggedness for telemetered Western Rattlesnakes in British Columbia. Trend lines (shown) were fit for the pooled group of snakes during the entire Outgoing migration (*R*^*2*^ = 0.13), the Initial migration (*R*^*2*^ = 0.09) and the Late stage of migration (*R*^*2*^ = 0.13). Forest snakes are represented by solid markers; Open-Habitat snakes are represented by open markers

In all three categories of the migration (Outgoing, Initial, Late) the observed thermal values derived from the migratory pathways of the Forest snakes were significantly higher than the same values for the Open-Habitat snakes, as compared to their respective simulated movements (Table 2 B, Fig. 4). These differences were most noticeable during the Late stage of migration, when the thermal values of the observed (observed) pathways for Forest snakes averaged near the 80th percentile, compared to a 50th percentile average for the snakes that remained in open habitats. In fact, thermal values for the Open-Habitat snake migrations averaged close to 50th percentile scores in all three migration categories (Table 2 B). The average probability values (from z-test scores) as determined for the Outgoing, Initial and Late stages of the migration were significantly lower for Forest snakes than Open-Habitat snake (Table 2 C).

The ruggedness index values for migration paths were significantly higher for Forest snakes (*x̄* =77.7 ± 23.0) than for Open-Habitat snakes (x̄ =56.6 ± 13.2; t_22_ = 3.09, *P* < 0.01). A significant effect of ruggedness was found for the percentile score of the observed migration path values for the Late migration category (F_1,28_ = 4.31, *P* = 0.047; *R*^*2*^ = 0.13), but not for either the Outgoing migration (F_1,28_ = 4.05, *P* = 0.054; *R*^*2*^ = 0.13) or Initial migration stages (F_1,28_ = 2.97, *P* = 0.095; *R*^*2*^ = 0.09; Fig. 3). Rattlesnake hibernacula with higher average ruggedness values were more likely to produce snakes that migrated to forest habitat (t_25_ = 3.99, *P* = < 0.001).

## Discussion

Overall, the results of our study indicate that the annual migrations of these northern snakes are dictated at least in part by thermal attributes of landscapes, at a relatively large-scale, particularly for snakes that utilize forested habitat. This is intuitive for terrestrial ectotherms occurring at high latitudes, but what is more interesting is the fact the animals appear to travel relatively longer distances to access this habitat, and their movements take them out of the lower arid grassland valleys that are considered typical summer habitat. This pattern also was not universal: exactly half of the animals we followed undertook the longer migrations into the higher elevation forests, and slightly greater than half of the animals travelled into relatively warmer areas on the landscape. Thus, the thermal parameters we examined in this study do not fully explain the dichotomy of movements exhibited by snakes in this and a previous study [29], but they do shed light on the factors influencing migration patterns in northern herpetofauna.

Use of forest habitat by rattlesnakes is well-documented but in locations further south and east than our study location [63, 81, 83]. Similarly, long-distance movements also have been reported elsewhere [4, 24, 45] and within the same region of our study [29]. Long-distance migratory movements for northern snakes, in general, have often been hypothesized to reflect widely separated resources, such as hibernacula and summer foraging sites. The mean migration distances in our study were similar to those previously reported in this region; however, several of the maximum distances recorded in this study were longer than those previously reported [5, 13, 14, 29, 49, 52]. The prevalence of forest habitat use [first detected by Gomez et al. [29] and now well-demonstrated by our study] is somewhat unexpected, given the presumption that the animals at their northern limits should be strongly tied to the arid, open grassland habitat of the valley bottoms [52, 55].

Our analysis partially explains the patterns of summer habitat use by these animals. The variable terrain of British Columbia coincides with the northern limit of the species, providing energetic and thermoregulatory challenges for the animals and raising the benefits of using habitat with optimal thermal attributes. In particular, the thermal landscape properties of the outgoing migration as a whole and the late stage of migration differed the most from the simulated random walks. During this time, most of the snakes travelled along warmer pathways, and snakes heading to or occupying forest habitats tended towards warmer pathways. Snakes appeared to move through a variety of conditions to reach destination habitats with ideal thermal properties, as evidenced by fewer snakes travelling warmer pathways during the initial migration stage. The consequences of using cooler paths are not known; however, it is likely that microhabitat selection may compensate for temperature changes [11, 77, 86].

Rattlesnakes migrating through landscapes with higher ruggedness (more elevation variation in the terrain) were more likely to use forested habitats and had higher migration path percentiles during the late stage of migration. Although this relationship was significant, the amount of variation actually attributable to ruggedness was low. Still, ruggedness has been included as an important attribute in habitat selection for a variety of wildlife including caribou [60], bighorn sheep [74], badger [1] and grouse [12], and our data support the assertion that this metric should be considered an important influence in snake habitat use [26, 30], at least in northern areas with noticeable variation in topography.

Thermal patterns of the landscape (as we measured them) appeared to be influencing the migrations observed in our study; however, there may be additional factors dictating migration routes that work in combination or separate from thermal attributes of the landscape. Animals may migrate in search of resources such as prey, mates or other suitable habitat conditions. Several studies link altered spatial behaviours to prey availability [23, 82], whereas others indicate limited support for this effect [61, 78]. In Wyoming, the movement of male rattlesnakes has been attributed to mate-searching [24]. There is, however, insufficient knowledge to extrapolate these effects to other locations, such as our northern study site. While our results indicate that there may be landscape thermal influences on snake movements, investigation of other putative factors is warranted.

As with all ecological models, thermal landscape simulations are simplistic representations of complex systems. The thermal models developed in this study provided insight into the role that thermal attributes of the landscape play in rattlesnake habitat use during the active season; however, they may be constrained by a spatial database resolution of only 25 × 25 m pixels. As the thermal landscape used in the analysis is based on this resolution, any variation occurring below this scale is not captured. A higher resolution digital elevation model, perhaps 3 × 3 m, in concert with a ground-cover mapping layer such as LIDAR, could be used to examine landscape dynamics through a finer lens.

The results of this study demonstrate that the relationship between hibernaculum location, migration distance and direction, and summer habitat utilization for these animals is far more complex than initially suspected. This is particularly important given our understanding of northern rattlesnake ecology historically has been based largely on one study that collected detailed information on one population of snakes [52]. Clearly, widespread migratory differences exist both within and between hibernating populations of these animals. To further our understanding of the ecology of migratory animals, we need to realize di- and polytomous habitat use may require a reassessment of “typical” habitat [18, 70].

Climate-induced shifts in ecosystem boundaries and thermal regimes, such as encroachment of forests into grassland habitats and warming climate, may have significant effects on the habitat use by reptiles, including the subjects of this study [43, 48]. The effects on northern snakes are likely two-fold: First, changes in the vegetation will modify the structure and heterogeneity of the landscape, requiring behavioral modifications to small and large-scale thermoregulation [46]. On a landscape scale, expansion of one habitat type (e.g. the bunchgrass zone in this study) could mean longer migrations to reach other habitat (forested habitat as detected in this study). This will lead to increased energy expenditure on travel, perhaps reducing overall fitness. This potentially will have significant impacts on reptile species that use these habitats in temperate regions [19].

## Conclusions

Although the thermal landscape seems to explain some variation with respect to forest migrants, grassland migrants selected pathways that seemed random with respect to the thermal landscape, at least at the resolution of our analysis. Still, this work provides new insight into the factors dictating seasonal snake movements in locations where extensive migrations occur. On a landscape scale, snakes use habitats that provide a thermal advantage through the short, northern summer. The role of thermal landscape attributes in colder environments, and how they affect migratory pathways of snakes (and other species) warrants consideration along with other resource values in assessing habitat. For northern snakes, developing a tool that precisely predicts summer movement patterns of snakes emanating from specific dens may present a lofty challenge, but any insight or mapping tool that can delineate probable movement pathways will greatly improve our ability to manage ecosystems. This may be particularly important where economic drivers of landscape use (e.g. ranching, forestry) coincide with high-value species habitat. Additionally, understanding local and landscape-scale movement patterns has obvious management applications for species likely to be affected by climate-induced shifts in ecosystem boundaries and thermal regimes.

## Data Availability

Digital elevation model and biogeoclimatic zone spatial data are available upon request from the BC Ministry of Forests, Land, Natural Resource Operations and Rural Development. Telemetry data are archived with the BC Conservation Data Centre as part of the Wildlife Species Inventory (WHI) managed by the BC Ministry of Environment.

## Abbreviations

GIS: Geographic Information System
SD: Standard Deviation
UTM: Universal Transverse Mercator
